# Social determinants of access to healthcare services by race/ethnicity in a Southeastern Metropolitan Area

**DOI:** 10.1080/29944694.2026.2635823

**Published:** 2026-03-27

**Authors:** Jonathan H. Low, Tony Vu, Joseph Keawe’aimoku Kaholokula, Eunjung Lim, Michael Cobo, Bhonesa Kirpal, Glynn Chatmon, Sarah B. Maness

**Affiliations:** a School of Business, College of Charleston, Charleston, South Carolina, USA;; b School of Medicine, Meharry Medical College, Nashville, Tennessee, USA;; c Department of Native Hawaiian Health, John A. Burns School of Medicine, University of Hawai’i at Manoa, Honolulu, Hawaii, USA;; d Department of Health and Human Performance, College of Charleston, Charleston, South Carolina, USA;; e Department of Health Education and Promotion, East Carolina University, Greenville, North Carolina, USA

**Keywords:** Access to healthcare, social determinants, health inequity, race/ethnicity

## Abstract

Ethnic and minority groups throughout the United States experience higher rates of sickness and death across a wide range of health conditions. The purpose of this study was to assess social determinants of healthcare access in the Charleston area by race/ethnicity. This study utilized a secondary data set from the 2019 Trident United Way Community Health Needs Assessment. Variables included self-reported reasons for limited healthcare access and race/ethnicity. Results indicated significant differences by race/ethnicity based on not knowing how to access health services (p=.034); not knowing when to see a doctor (p=.006); fear (p=.015); and lack of trust of doctors (p=.014). Additional significant differences were found for cultural/religious beliefs; health insurance related barriers; lack of transportation; and lack of knowledge (all p<.00l). Findings indicate that disparities exist by race/ethnicity related to reasons for limited healthcare access in the Charleston Tri-County area.

## Introduction

Ethnic and minority groups throughout the United States experience higher rates of sickness and death across a wide range of health conditions. A representative national survey indicated that Black individuals with low income had the highest prevalence of poor or fair health (24.9%) while White individuals with middle or high income had the lowest (6.3%) (Mahajan et al., 2021).

Racial/ethnic minority groups are less likely to receive preventive health services and often have increased health disparities due to a history of systemic racism (Jones et al., 2022; Jung et al., 2016). The theoretical framework Jones’ Levels of Racism posits that racism can be understood on three levels, institutionalized, personally mediated, and internalized (Jones, 2000). Institutionalized racism is differential access to goods, services and opportunities of society based on race. This type of racism aligns with the Social Determinants of Health (SDOH) as it manifests in differential access to education, housing, employment, healthcare and environment. Institutionalized racism has and continues to influence access to healthcare through factors such as implicit bias of healthcare providers, economic inequality, and a distrust in the healthcare system rooted in a history of mistreatment (Maness et al., 2021).

Healthy physical, social, and economic environments strengthen the potential to achieve health and well-being. Addressing SDoH and institutionalized racism has the potential to eliminate health disparities and improve health and well-being of all” (U.S. Department of Health & Human Services, 2020b). The Healthy People 2030 SDOH Framework outlines how key areas impact health, including education, neighborhood, social and community context, economic stability, and healthcare access (U.S. Department of Health & Human Services, 2020a). The focus of the current manuscript is access to healthcare.

Despite an overall increase in insurance coverage in the Tri-County Charleston Metropolitan area (Tri-County Area) in South Carolina, the number of premature deaths increased between the years of 2015 to 2020 and racial and ethnic disparities persist. (County Health Rankings, 2024). In a qualitative study examining access to healthcare among African American adults in the Charleston participants indicated problems with appointment availability, expense of care, lack of transparency in insurance coverage, the need for more primary care clinics and enhanced community outreach and education on how to access healthcare (Maness et al., 2023).

The aim of this project was to utilize a Social Determinants of Health approach to investigate differences in access to healthcare by race/ethnicity.

## Methods

This study utilized the 2019 Community Health Needs Assessment Report conducted by Trident United Way in conjunction with the Medical University of South Carolina and Roper St. Francis Hospital.

### Study design

Partnering with Trident United Way (TUW), a charity organization that promotes health equity in Charleston, SC, we utilized their Community Health Needs Assessment (CHNA) of the population in the Tri-County area. The aims of this project were to investigate differences in access to healthcare by race/ethnicity, assessing self-reported access to healthcare services of those who were insured and uninsured in the year of 2019 in the Tri-County Area.

### Participants

Participant criteria included individuals over the age of 18 living in the Tri-County area of Charleston, Berkeley and Dorchester counties in South Carolina.

### Measures

Trident United Way provided a dataset from the 2019 Community Health Needs Assessment Survey. The dataset provided by Trident United Way from the 2019 Community Health Needs Assessment Survey included demographic questions and a series of questions designed to assess barriers to healthcare access. Demographic information collected included race/ethnicity, gender, age, education level, income, employment status, residential area, self-reported overall health, and insurance type. Race/ethnicity was categorized as White, Hispanic, Black, and Other. Gender, education, income, and employment were assessed using standard categorical options, while residential area and self-reported health were collected through open-ended responses or predefined categories relevant to the Tri-County area.

All questions assessing barriers to healthcare access had response options of “Yes” or “No.” For cultural and religious beliefs, participants were asked, “Do your cultural or religious beliefs prevent or limit your access to healthcare services?” To evaluate knowledge of healthcare access, participants were asked, “Do you know how to access healthcare services when needed?” Awareness of the need to see a doctor was assessed by asking, “Do you know when it is necessary to see a doctor?” Participants were also asked about fear as a potential barrier with the question, “Does fear prevent you from accessing healthcare services?” The survey addressed trust issues with the question, “Do you trust doctors to provide you with the care you need?” Questions about financial and logistical barriers included, “Do you have health insurance?” and “If you have insurance, can you afford the co-pays or deductibles required for care?” Additionally, participants were asked, “Do you know how to sign up for health insurance?” to assess knowledge barriers. Other logistical barriers were captured with questions like “Do you lack transportation to healthcare services?” and “Does the distance to healthcare services make it difficult for you to access them?” Finally, participants were asked about general knowledge with the question, “Do you feel you have enough information to make informed decisions about your healthcare?”

### Data processing and sample selection

This study utilized the 2019 Community Health Needs Assessment (CHNA) dataset provided by Trident United Way (TUW), initially comprising 5,128 respondents. The CHNA data analysts used weighting procedures to adjust for sampling bias and to better reflect the demographics of the Tri-County area. To calculate weights, they combined survey demographic data with U.S. Census population estimates from the American Community Survey (U.S. Census Bureau, 2025). They trained a Random Forest model to predict the probability that each case in the combined dataset belonged to the survey group, then took predicted propensity scores for cases from the CHNA survey and calculated weights as 1/propensity score.

To ensure data quality and relevance, we applied specific inclusion and exclusion criteria, removing respondents under 18 years of age (n = 28) per the study’s focus on adults, as well as those with missing data for key variables such as race/ethnicity (n = 99), age (n = 57), and other demographic or outcome variables (approximately 29% missing responses for reasons for limited healthcare access). The final analytical sample consisted of 3,364 participants. Race/ethnicity categories were consolidated into four groups—White, Black, Hispanic, and Other—by combining smaller groups (e.g., American Indian/Alaska Native, Asian, Native Hawaiian/Pacific Islander, Other/Multi-Racial) into the “Other” category. These racial/ethnic groups were collapsed into an “Other” category due to small sample size and to protect the potential identification of participants. Percentages reported in Tables 1 and 2 are weighted using the CHNA post-stratification weight to ensure representativeness of the Tri-County Charleston Metropolitan area population, while sample sizes (N) reflect unweighted counts.

### Data analysis plan

Variables analyzed were self-reported reasons for limited healthcare access by race and ethnicity. Researchers used Rao-Scott Chi Square tests in excel for all analyses. The Rao-Scott chi square test is a design-adjusted version of the Pearson chi-square test (Rao & Scott, 1979). It detects whether there is a significant association between categorical variables while accounting for the complex survey design.

## Results

Most of the study participants (n = 3364) were White, with Hispanic and Other groups making up the smallest percentages (Table 1). Most participants were female (75.2%), employed full time (67.2%), had private insurance (59.7%) and reported their overall health as good (40.6%) or very good (29.6%). Regarding age, there was a relatively even distribution of participants between 18 and 65 years across racial groups.

Significant differences existed by race/ethnicity for each demographic category except for gender (p = 0.744). Black and Hispanic participants had the highest percentages of individuals with less than a high school education (7.2% and 23.2%, respectively), while White and Other groups had the highest percentages with an associate degree or higher. Similarly, income disparities were evident, as White and Other groups had the highest percentages of participants earning $50,000 or more, while Black and Hispanic groups predominantly fell into lower income brackets, particularly under $30,000. Black and Hispanic participants were most likely to report earning under $30,000 per year (49.4%, 54.9%). Black and Hispanic participants and least likely to report working full time (55.1%, 52.5%) in comparison with White participants (74.3%) Stratified by race, Hispanic participants were most likely to report no insurance coverage (36.9%). White participants had the highest rate of private insurance coverage (69.7%). Black participants were most likely to report using public insurance (30.3%). Residential patterns showed that White participants were more concentrated in Berkeley (46.7%), while Black participants were predominantly in Charleston (48.0%).

Statistically significant differences were observed between race/ethnicity for most reasons for limited healthcare access, including cultural/religious beliefs, fear, not trusting doctors, lack of insurance, insufficient insurance, lack of transportation, work schedule, and lack of knowledge. Cultural and religious beliefs were identified as a significant barrier, with Hispanic participants (4.4%) reporting this issue at a higher rate than White (0.2%), Black (1.1%), and Other (0.6%) participants (p < 0.001). Fear as a barrier to healthcare was most frequently reported by White participants (4.3%), followed by Other (3.0%), Hispanic (2.3%), and Black (2.1%) participants (p = 0.015).

Distrust of doctors was reported most often by Hispanic participants (3.0%), followed by Other (1.9%), White (2.1%), and Black (0.6%) participants (p = 0.014). Regarding insurance-related barriers, the inability to access or afford healthcare due to lack of insurance was significantly more common among Hispanic participants (19.1%) than White (5.2%), Black (6.1%), and Other (9.8%) participants (p < 0.001). Additionally, the inability to afford co-pays or deductibles was a significant barrier for White participants (15.5%), followed by Other (10.6%), Black (6.9%), and Hispanic (3.6%) participants (p < 0.001).

Transportation barriers were most prevalent among Black (4.5%) and Other (4.7%) participants, compared to White (1.6%) and Hispanic (0.8%) participants (p < 0.001). Work schedule conflicts were a significant barrier for White participants (13.7%), followed by Other (13.4%), Black (9.3%), and Hispanic (6.0%) participants (p < 0.001). Additionally, lack of knowledge was a prominent barrier for Hispanic participants (12.2%), compared to Other (4.6%), Black (3.1%), and White (2.8%) participants (p < 0.001). No significant differences were found for barriers related to “lack of available healthcare services” (p = 0.415) and “distance to healthcare services or unsafe location” (p = 0.451).

## Discussion

The findings of this study underscore the significant racial and ethnic disparities in the social determinant area of healthcare access within the Charleston Tri-County area, with economic barriers being the most prominent for minority populations.

Hispanic participants reported higher levels of cultural or religious beliefs as a barrier. This suggests that cultural factors may disproportionately impact Hispanic individuals’ healthcare access, necessitating culturally tailored interventions to address these challenges. Additionally, the Hispanic population reported higher levels of distrust in doctors This finding highlights the importance of building trust, particularly in Hispanic communities, where skepticism toward the medical establishment could hinder healthcare engagement. These challenges may be exacerbated by factors such as language barriers and recent immigration status, which complicate their ability to navigate the healthcare system. Immigrant populations often face significant challenges in accessing care, including language barriers, unfamiliarity with healthcare systems, and discrimination, leading to lower levels of trust in medical professionals (Kaczynski et al., 2025; National Academy of Sciences, 2016). This suggests the need for targeted interventions that address not only the financial and logistical barriers but also the cultural and linguistic differences that affect immigrant communities. Tailored programs aimed at improving healthcare literacy, building trust, and providing language support could be crucial for addressing these disparities. Initiatives specific to the Charleston area could include cross-sector partnerships with organizations such as the local Hispanic Cultural Association and Hispanic-serving religious congregations.

Hispanic participants were most likely to report no insurance coverage and Black participants were most likely to report using public insurance. These differences suggest challenges for minority groups in accessing private insurance, highlighting the disproportionate burden of being uninsured for Hispanic individuals. Private health insurance typically offers several advantages over public insurance, including broader access to healthcare providers, shorter wait times, and coverage for a wider range of medical services. Studies show that private plans often provide access to specialists without requiring referrals, more comprehensive prescription drug coverage, and additional benefits such as dental and vision care (Bhavsar et al., 2022). Moreover, individuals with private insurance experience less restrictive network limitations, allowing them greater flexibility in choosing providers and facilities. These benefits contribute to improved health outcomes and higher patient satisfaction, as private insurance plans often emphasize preventative care and wellness programs (National Academy of Medicine, 2021).

In contrast, public insurance programs like Medicaid and Medicare are designed to ensure access to essential healthcare services for vulnerable populations, including low-income individuals, older adults, and people with disabilities. While these programs reduce financial barriers to healthcare, they are often associated with longer wait times, limited provider networks, and fewer coverage options for non-essential services (Kaiser Family Foundation, 2023). The analysis revealed that Hispanic participants, who had the highest rate of being uninsured (36.9%), likely face significant challenges in accessing the benefits associated with private insurance. Similarly, Black participants, who reported the greatest reliance on public insurance (30.3%), may encounter limitations in coverage scope and provider availability. Local initiatives to reduce health inequities could include a community-focused navigator program to help community members access and understand their health insurance options.

White participants were more likely than other racial/ethnic groups to report having insurance but not being able to pay the deductible, even though they reported higher income and education levels. This may be influenced by the finding that White participants were more much more likely to have private insurance overall. Cost related medical debt is more common among people with private insurance they are more likely to have higher out of pocket costs than those in public plans (Allen et al., 2021; Wray et al., 2023). This finding challenges the assumption that higher income directly translates into better healthcare access, revealing that even those with insurance may face financial barriers when out-of-pocket costs are prohibitively high. This aligns with previous studies, such as that by Bhavsar et al. (2022), which found that financial barriers, including high co-pays and deductibles, were key determinants of limited healthcare access, even among insured populations (Bhavsar et al., 2022).

Transportation barriers were most prevalent among Black participants, suggesting that transportation infrastructure or alternative solutions may be especially needed in communities of color to improve healthcare access. Previous work has indicated that racial inequities in healthcare visits between Black and White patients were amplified by transportation barriers (Maleki & Smith-Colin, 2025). One recent South Carolina that used Structural Equation Modeling to identify factors that influence healthcare access found both African American and Hispanic respondents had significantly higher concerns about transportation safety in relation to accessing healthcare (Ning et al., 2025). While transportation safety may not immediately seem to relate to healthcare access, minority populations are more likely to live in communities with less pedestrian friendly neighborhoods and more likely to rely on walking or public transportation to access healthcare. Charleston ranks among the top 10 most dangerous cities for pedestrians, yet organizations such as Charleston Moves are working to improve safety for biking walking and public transportation (Smart Growth America, 2025, Charleston Moves, 2025).

Hispanic participants were most likely to report lack of knowledge as a barrier. This includes issues such as not knowing how to access services, when to seek care, or how to sign up for insurance, indicating a need for targeted educational programs to address these knowledge gaps, particularly in Hispanic communities. Interestingly, no significant differences were found concerning the availability or distance to healthcare services, pointing to the fact that other factors—such as affordability and cultural competency—may be more significant barriers to care than geographic access alone. This aligns with findings from The Commonwealth Fund (2020), which suggests that factors like insurance coverage, trust in providers, and cultural competence are more influential in healthcare access than proximity to healthcare facilities.

White participants were most likely to report work schedule as a barrier to healthcare. Our demographic findings indicate that White participants had the highest percentage of full time work (74.3%) which may influence these results. Previous work has indicated that time pressure related to work obligations is associated with barriers to obtaining preventative healthcare (Yao et al., 2015). Health appointments are often available during standard working hours and more flexible appointment options may help mitigate this issue. Studies have indicated that telehealth appointments have had higher adherence to attending appointment than in person, with few missed appointments among White and Asian patients (Adepoju et al., 2022; Snoswell & Comans, 2021). Additionally,

White participants were also most likely to report fear as a barrier, which is interesting as fear is often intertwined with medical mistrust, for which reports were lower among this group. Many studies examining fear as a barrier to healthcare have focused on discrimination among minoritized populations (Hacker et al., 2015; Seelman et al., 2017). Future research should examine the differences between medical distrust and fear and assess reasons for fear and distrust across multiple race/ethnicities, including White participants. Additionally, many recent studies about fear and the healthcare system focus on the COVID-19 pandemic, which occurred after data collection for the current study (Pujolar et al., 2022; Vázquez et al., 2024).

Overall, addressing identified disparities requires support in accessing and navigating health insurance. This may include local community efforts to connect community members with existing resources, as well as expanding affordable private insurance options or enhancing public insurance programs to close coverage gaps. Peer health navigator and community health worker models are a feasible way to address social determinants of health in a wide range of settings (Foster et al., 2023; Valeriani et al., 2022). Community Health Workers are trusted members of the communities they serve and are trained facilitate access to health services (Knowles et al., 2023). In Charleston, academic and community partnerships such as the Community Engagement Program at the South Carolina Clinical and Translational Research Institute could be leveraged to create effective local interventions (Medical University of South Carolina, 2023). The Community Engagement Program strives to engage community members and academic partners in all parts of the research process to improve health and provides consultative services and a research training program to researchers and community partners.

As healthcare costs continue to rise, ensuring equitable access to care for all populations will depend on reforms that improve affordability, particularly for those in lower-middle-income brackets. A study by Sommers et al. (2017) found that expanding Medicaid eligibility significantly reduced uninsured rates and improved access to care, especially among low-income and minority populations. Expanding public health programs, such as Medicaid, and introducing policies that reduce the financial burden of co-pays and deductibles could alleviate some of the economic barriers faced by uninsured and underinsured populations. At the same time, enhancing private insurance options to include more affordable plans would benefit individuals who are employed but still struggle to cover high out-of-pocket costs. According to The Commonwealth Fund (2020), expanding affordable private insurance options would help reduce the financial strain on working individuals who earn too much to qualify for public programs like Medicaid but still face high out-of-pocket costs. Ensuring that insurance options are not only affordable but also comprehensive is key to reducing healthcare disparities and improving the quality of care for marginalized groups. Additionally, creating a more culturally competent healthcare system that acknowledges the diverse needs of underrepresented populations can help improve patient trust, adherence to treatment plans, and overall health outcomes. As noted by Betancourt et al. (2005), culturally competent care improves patient satisfaction, treatment adherence, and health outcomes, especially for ethnic and racial minorities.

This study has several limitations. First, it relied on voluntary, self-reported data, which is subject to response and selection bias. Results may not be representative of the larger population. This study used secondary data, limiting the range of SDoH variables available or use of a framework. Participants may have underreported or misrepresented their healthcare access barriers due to social desirability bias or limited understanding of healthcare concepts. Additionally, the study sample was restricted to residents of the Charleston Tri-County area, which may limit the generalizability of the findings to other regions with different demographic profiles. This study used a cross-sectional design which prevents assessing participants responses over time. Due to small sample size, several categories of racial/ethnic groups (American Indian/Alaska Native, Asian, Native Hawaiian/Pacific Islander, Other/Multi-Racial) were collapsed into one group (Other) for analyses, preventing us from identifying potentially important disparities within each group. While this study focused primarily on race/ethnicity, other factors, such as gender, sexual orientation, and disability status, could also play important roles in healthcare access and should be explored in future studies. Additionally, despite weighting procedures, there was overrepresentation of higher-income, White participants that may have biased our results and limited the generalizability of our study. Furthermore, the data were collected prior to the COVID-19 pandemic, which significantly impacted disparities in healthcare access. As such, our findings cannot be generalized to that time-period nor do they address the sustained impacts post-COVID. This study analyzed barriers separately and is not able to make determinations on interaction effects between variables. Lastly, because the study was cross-sectional, it does not establish causal relationships between race/ethnicity and healthcare access barriers.

Future research should explore social determinants of access to healthcare further, particularly through qualitative studies that capture the nuances of healthcare access challenges in the Tri-County Charleston area. Future research should also consider longitudinal studies to track changes in healthcare access over time, evaluating the effectiveness of interventions designed to reduce disparities. These could include the impact of policy changes, such as the expansion of Medicaid, or local community health initiatives aimed at improving the affordability and accessibility of care.

## Conclusion

In conclusion, these findings highlight the multifaceted nature of barriers to healthcare access, with distinct differences across racial and ethnic groups in the Tri-County Charleston area. Leveraging cross-sector community partnerships to address cultural beliefs, fear, knowledge gaps, as well as social determinants including transportation issues and insurance gaps is crucial for reducing disparities and improving healthcare equity. Expanding affordable private insurance options or enhancing public insurance programs to close coverage gaps and improve healthcare quality could help alleviate these inequities. Ensuring equitable access to healthcare requires targeted interventions that consider the diverse needs of underrepresented populations.

## Figures and Tables

**Table 1.** Demographic information.

	Total	White	Black	Hispanic	Other	
Variable	(N = 3364)	(N = 1995; 61.5%)	(N = 987; 27.8%)	(N = 215; 6.0%)	(N = 167; 4.7%)	P-value
**Gender**						0.744
Female	2745 (75.2%)	1613 (74.9%)	824 (76.1%)	174 (72.9%)	134 (74.9%)	
Male	591 (24.5%)	368 (24.7%)	161 (23.8%)	37 (27.1%)	25 (24.7%)	
Transgender	8 (0.3%)	6 (0.3%)	1 (0.1%)	0 (0%)	1 (0.3%)	
**Age**						<0.001
18–24	167 (4.9%)	84 (4.2%)	55 (5.4%)	17 (7.9%)	11 (6.9%)	
25–34	648 (18.0%)	359 (17.0%)	184 (17.2%)	73 (32.7%)	32 (18.2%)	
35–44	887 (26.1%)	556 (27.5%)	209 (20.8%)	82 (37.9%)	40 (24.0%)	
45–54	687 (20.6%)	450 (22.5%)	171 (17.6%)	26 (12.9%)	40 (23.1%)	
55–64	622 (18.9%)	362 (18.8%)	219 (22.3%)	12 (5.9%)	29 (18.0%)	
>64	353 (11.5%)	184 (10.1%)	149 (16.8%)	5 (2.8%)	15 (9.8%)	
**Education**						<0.001
Less than High School	176 (5.4%)	53 (2.8%)	65 (7.2%)	49 (23.2%)	9 (5.7%)	
High School or Some College	1281 (37.9%)	645 (32.2%)	478 (49.0%)	85 (40.4%)	73 (42.6%)	
Bachelor’s or associate Degree	1227 (36.8%)	810 (40.8%)	300 (29.8%)	57 (28.6%)	60 (37.1%)	
Graduate Degree	662 (19.9%)	482 (24.2%)	138 (13.9%)	17 (7.8%)	25 (14.6%)	
**Income**						<0.001
Under $30,000	869 (28.3%)	285 (16.0%)	440 (49.4%)	92 (54.9%)	52 (35.9%)	
$30,000–$49,999	524 (17.1%)	278 (15.2%)	191 (21.0%)	32 (19.0%)	23 (16.4%)	
$50,000–$74,999	489 (16.4%)	341 (18.8%)	112 (12.5%)	12 (7.6%)	24 (16.8%)	
$75,000–$99,999	416 (14.3%)	316 (17.8%)	70 (8.1%)	13 (8.1%)	17 (11.3%)	
Over $100,000	690 (23.9%)	572 (32.1%)	76 (9.0%)	16 (10.5%)	26 (19.5%)	
**Employment**						<0.001
Full-time employed	2086 (67.2%)	1381 (74.3%)	537 (55.1%)	72 (52.5%)	96 (62.3%)	
Part-time employed	307 (9.5%)	139 (7.2%)	113 (11.8%)	35 (22.9%)	20 (11.6%)	
Not employed/Disabled	371 (11.9%)	149 (8.4%)	174 (18.5%)	30 (18.1%)	18 (11.9%)	
Retired	262 (9.1%)	137 (8.1%)	112 (12.5%)	2 (1.2%)	11 (7.7%)	
Student	79 (2.4%)	38 (2.0%)	22 (2.1%)	8 (5.4%)	11 (6.6%)	
**Residential area**						<0.001
Berkeley	1397 (40.6%)	961 (46.7%)	291 (28.2%)	82 (37.6%)	63 (35.7%)	
Charleston	1369 (40.3%)	714 (35.9%)	479 (48.0%)	103 (47.8%)	73 (43.8%)	
Dorchester	362 (12.2%)	213 (12.0%)	112 (12.8%)	15 (9.4%)	22 (15.8%)	
Other	211 (6.9%)	101 (5.4%)	92 (11.0%)	10 (5.3%)	8 (4.7%)	
**Self-reported overall health**						<0.001
Poor	97 (3.3%)	44 (2.6%)	38 (4.5%)	9 (4.4%)	6 (4.1%)	
Fair	502 (16.2%)	256 (14.0%)	170 (19.2%)	49 (22.5%)	27 (17.8%)	
Good	1273 (40.6%)	709 (38.8%)	397 (42.4%)	102 (48.3%)	65 (43.5%)	
Very Good	905 (29.6%)	602 (33.2%)	230 (25.0%)	37 (18.4%)	36 (25.3%)	
Excellent	315 (10.3%)	207 (11.4%)	82 (8.9%)	13 (6.3%)	13 (9.3%)	
**Insurance**						<0.001
No Insurance	323 (9.6%)	132 (6.8%)	91 (9.8%)	80 (36.9%)	20 (11.2%)	
Other	495 (14.8%)	269 (13.6%)	142 (14.5%)	55 (26.5%)	29 (17.5%)	
Private	2003 (59.7%)	1400 (69.7%)	456 (45.3%)	51 (24.3%)	96 (58.1%)	
Public	542 (15.9%)	194 (10.0%)	297 (30.3%)	29 (12.3%)	22 (13.3%)	

N = sample size.

P<.05 = statistical significance.

Unweighted N (Weighted column %).

Rao-Scott chi-square test was used to explore bivariate association with race/ethnicity.

**Table 2.** Reason for limited healthcare access by race/ethnicity.

Reason	Total	White	Black	Hispanic	Other	P-value
Cultural/religious beliefs	0.7*	0.2	1.1	4.4	0.6	<0.001
Fear	3.5	4.3	2.1	2.3	3.0	0.015
I do not trust doctors	1.7	2.1	0.6	3.0	1.9	0.014
Lack of available health care services	2.5	2.6	1.9	2.8	3.8	0.415
I do not have insurance and cannot pay for services	6.5	5.2	6.1	19.1	9.8	<0.001
I have insurance, but I cannot afford the co-pay or deductibles	12.1	15.5	6.9	3.6	10.6	<0.001
Lack of transportation	2.5	1.6	4.5	0.8	4.7	<0.001
Work schedule	12.0	13.7	9.3	6.0	13.4	<0.001
Health services are too far away or not in a safe part of town	1.0	1.1	0.9	0.0	1.0	0.451
Lack of Knowledge	3.5	2.8	3.1	12.2	4.6	<0.001
Other	3.5	3.8	3.4	1.6	2.9	0.376

* Weighted %.

Rao-Scott chi-square test was used to explore bivariate association with race/ethnicity.

P<.05 = statistical significance.
